# Distribution of nickel and chromium containing particles from tattoo needle wear in humans and its possible impact on allergic reactions

**DOI:** 10.1186/s12989-019-0317-1

**Published:** 2019-08-27

**Authors:** Ines Schreiver, Bernhard Hesse, Christian Seim, Hiram Castillo-Michel, Lars Anklamm, Julie Villanova, Nadine Dreiack, Adrien Lagrange, Randolph Penning, Christa De Cuyper, Remi Tucoulou, Wolfgang Bäumler, Marine Cotte, Andreas Luch

**Affiliations:** 1 Department of Chemical and Product Safety, German Federal Institute for Risk Assessment (BfR), Max-Dohrn-Strasse 8-10, 10589 Berlin, Germany; 2 The European Synchrotron, CS 40220, 38043 Grenoble Cedex 9, France; 3 Xploraytion GmbH, Bismarckstrasse 10-12, 10625 Berlin, Germany; 4 Department of X-ray Spectrometry, Physikalisch-Technische Bundesanstalt, Abbestrasse 2-12, 10587 Berlin, Germany; 5 Institute for Optics and Atomic Physics, Technical University Berlin, Hardenbergstrasse 36, 10623 Berlin, Germany; 6 Helmut Fischer GmbH Institut für Elektronik und Messtechnik, Industriestrasse 21, 71069 Sindelfingen, Germany; 7 Institute of Materials Science and Technologies, Technical University Berlin, Strasse des 17. Juni 135, 10623 Berlin, Germany; 8 Institute of Forensic Medicine, Ludwig-Maximilians University, Nussbaumstrasse 26, 80336 Munich, Germany; 9 Dermatology, Meiboomstraat 15, Blankenberge, 8370 Belgium; 10 Department of Dermatology, University of Regensburg, Franz Josef Strauß Allee 11, 93042 Regensburg, Germany; 11 Laboratory of Molecular and Structural Archaeology (LAMS), Sorbonne University, CNRS, UMR8220, Paris, France

**Keywords:** Metallic wear, Nickel, Tattoo, Titanium dioxide, Synchrotron, XRF, Allergy

## Abstract

**Background:**

Allergic reactions to tattoos are amongst the most common side effects occurring with this permanent deposition of pigments into the dermal skin layer. The characterization of such pigments and their distribution has been investigated in recent decades. The health impact of tattoo equipment on the extensive number of people with inked skin has been the focus of neither research nor medical diagnostics. Although tattoo needles contain high amounts of sensitizing elements like nickel (Ni) and chromium (Cr), their influence on metal deposition in skin has never been investigated.

**Results:**

Here, we report the deposition of nano- and micrometer sized tattoo needle wear particles in human skin that translocate to lymph nodes. Usually tattoo needles contain nickel (6–8%) and chromium (15–20%) both of which prompt a high rate of sensitization in the general population. As verified in pig skin, wear significantly increased upon tattooing with the suspected abrasive titanium dioxide white when compared to carbon black pigment. Additionally, scanning electron microscopy of the tattoo needle revealed a high wear after tattooing with ink containing titanium dioxide. The investigation of a skin biopsy obtained from a nickel sensitized patient with type IV allergy toward a tattoo showed both wear particles and iron pigments contaminated with nickel.

**Conclusion:**

Previously, the virtually inevitable nickel contamination of iron pigments was suspected to be responsible for nickel-driven tattoo allergies. The evidence from our study clearly points to an additional entry of nickel to both skin and lymph nodes originating from tattoo needle wear with an as yet to be assessed impact on tattoo allergy formation and systemic sensitization.

**Electronic supplementary material:**

The online version of this article (10.1186/s12989-019-0317-1) contains supplementary material, which is available to authorized users.

## Background

Delayed type IV allergies triggered by tattoos constitute the second most reported side effects referred in the literature [1] and, e.g., account for 37% of all patients in a Danish tattoo clinic [2]. Combined with the prevalence of tattooed individuals—ranging from 8.5 to 24% of the population across Europe and the USA [3]—this shows that a tremendous number of people are affected. Past case reports identify chromium (Cr) [4], mercury (Hg) [5], cobalt (Co) [6] and nickel (Ni) [7] as sources of element-related allergies triggered by tattoos. Cleavage of organic pigments by UV or laser irradiation displays another source of carcinogens or sensitizers [8]. Analyses of tattoo inks frequently reveal a variety of carcinogenic and sensitizing elemental impurities such as Ni, Cr, Co or Hg that are mostly introduced by pigment particles [9]. It has also been occasionally speculated that metal exposure might derive from the tattoo needle used to implant the tattoo pigment into the skin [10].

Tattoo pigments including impurities are transported to the draining lymph nodes and likely to other organs either passively or by active transport through phagocytizing cells [11, 12]. In the lymph node, presentation of allergens to immune cells might result in sensitization. Mobility and kinetics of pigments including such sensitizers are highly dependent on particle size. In a previous study on the biokinetics of tattoo pigments in human tissue samples we found indications of preferred transport of smaller particles of an organic pigment from the skin to regional lymph nodes [13]. The average particle size in tattoo inks may vary from < 100 nm to > 1 μm [9, 14, 15]. The increased hazard of nano- compared to micro-sized particles is due to their increased surface-to-volume ratio, which consequently leads to a potentially higher release of toxic elements, if present. Additionally, nanoparticles in general can directly enter cells [16]. Smaller particles are more easily distributed, but may also be more easily excreted from the body [17]. However, little data exist on tattoo pigment sizes in different human tissues.

In this study, synchrotron-based nano-X-ray fluorescence (XRF) was used to analyze human skin and corresponding draining lymph nodes with the aim to establish a database of pigment particle sizes and elemental contaminants deposited in the human body. Data from the skin and lymph nodes of five donors with no known tattoo-related health effects were analyzed and compared to a skin biopsy of a patient with a type IV tattoo allergy and confirmed Ni sensitivity. Partly metallic characteristics of Ni and Cr in iron (Fe) particles were identified by means of synchrotron nano X-ray absorption near-edge structure (XANES) in the tissue specimen, which led us to investigate steel wear from tattoo needles and its subsequent quantification using *postmortem* tattooed pig skin.

## Results

### Nano- and micrometer sized metal particles in skin and lymph nodes

**Fig. 1:**
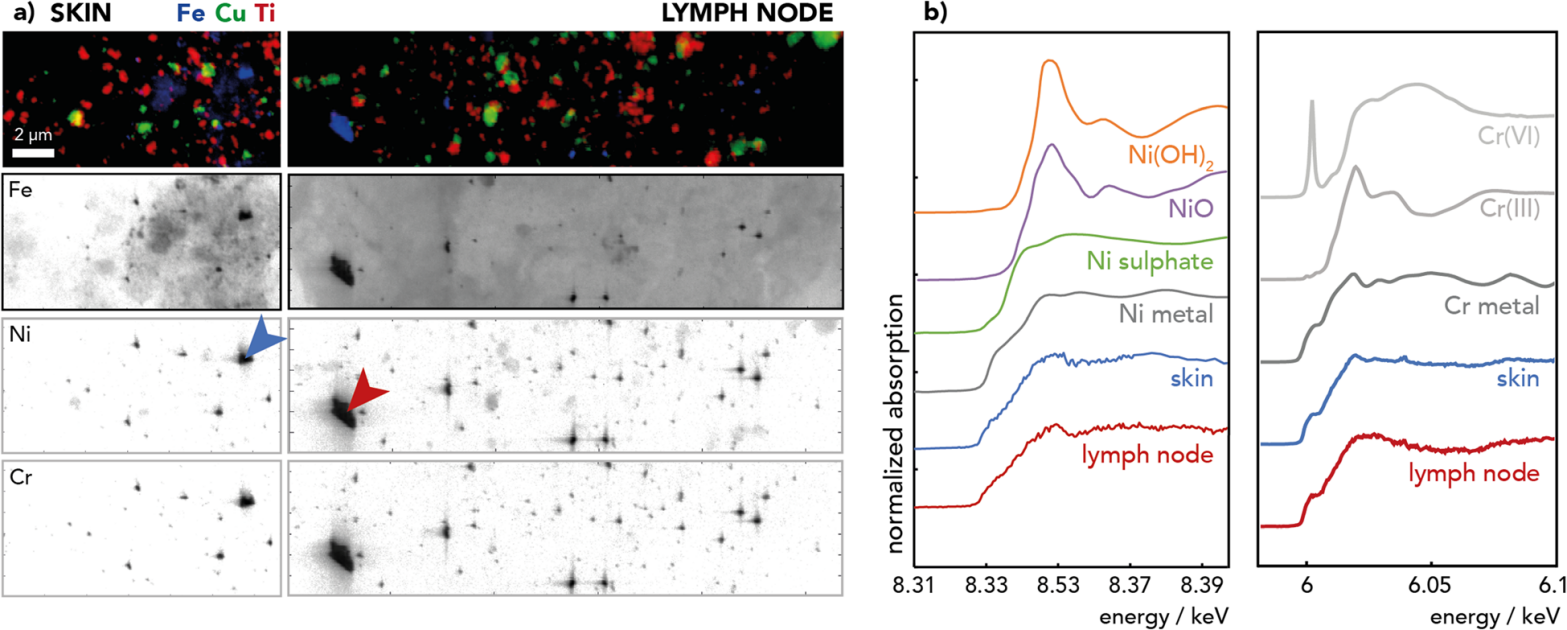
Synchrotron nano-X-ray fluorescence (XRF) and nano- X-ray absorption near-edge structure (XANES) analysis of representative skin and lymph node sections. **a** XRF elemental maps of donor 1 of titanium (Ti) from titanium dioxide, copper (Cu) from copper phthalocyanine, iron (Fe), chromium (Cr) and nickel (Ni) from steel debris, acquired at beamline ID16B with a resolution of 50 nm. Arrows point the regions where XANES spectra were acquired. **b** Cr and Ni K-edge XANES spectra of skin and lymph node samples mainly show metal Ni and a mixture of metal Cr(0) and ionic Cr (III) when compared to reference spectra

Tattoo particles in human skin and lymph node sections from deceased donors were analyzed by means of synchrotron nano-XRF to determine the particle size and elemental composition (Fig. 1a, Table 1, Additional file 1: Figure S1). Samples were selected with a special focus on bright colors, such as green, blue, and red, to obtain samples in which use of organic pigments mixed with inorganic white titanium dioxide (TiO_2_) was presumed. All exogenous elements detected in the skin appear to be transported to the corresponding draining lymph nodes, without significant modification of their size. Particle sizes ranged from 50 nm or smaller (resolution limit) up to a micrometer and depended on the particles elemental composition.

**Table 1:** XRF evaluation of particle sizes and elemental co-localization in skin and lymph node tissues from human donors (corpses)

Sample	NP size range in nm	Co-localizations
Donor 1 skin	Fe-Cr-Ni particles (50–1000), Cu (100–700), Ti (100–500), Fe (150–300)	yes: Fe-Cr-Ni, no: some Fe particles w/o Ni
Donor 1 LN	Fe-Cr-Ni particles (50–1000), Cu (100–1000), Ti (100–500)	yes: Fe-Cr-Ni no: Cu, Fe, Ti
Donor 2 skin left	Fe-Cr-Ni particles (50–500), Cu (100–250), Ti (200–250)	yes: Fe-Cr-Ni no: Cu, Fe, Ti
Donor 2 LN left	Fe-Cr-Ni particles (50–4000), Cu (100–250), Ti (200–250),	yes: Fe-Cr-Ni no: Cu, Fe, Ti
Donor 2 skin right	Fe-Cr-Ni particles (50–1400), Cu (100–300), Ti (200–300)	yes: Fe-Cr-Ni no: Cu, Fe, Ti
Donor 2 LN right	Fe-Cr-Ni particles (50–1500), Cu (100–300), Ti (200–300)	yes: Fe-Cr-Ni no: Cu, Fe, Ti
Donor 3 skin red	Fe-Cr-Ni particles (50–350), Cu (100–300), Ti (200–300)	yes: Fe-Cr-Ni no: Cu, Fe, Ti
Donor 3 skin green	Fe-Cr-Ni particles (100–650), Cu (100–800), Ti (200–400)	yes: Fe-Cr-Ni no: Cu, Fe, Ti
Donor 3 LN	Fe-Cr-Ni particles (50–500), Cu (100–300), Ti (200–300)	yes: Fe-Cr-Ni no: Cu, Fe, Ti
Allergy biopsy	Fe-Cr-Ni particles (50–300), Fe (300–450), Cu (100–1400), Ti (200–300)	yes: Fe-Cr-Ni no: Cu, Fe, Ti

*Abbreviations*: *LN* lymph node, *NP* nanoparticle, *Fe* iron, *Cr* chromium, *Ni* nickel, *Ti* titanium, *Cu* copper

Ti particles found in all tattooed samples derive from TiO_2_ (mixture of anatase and rutile crystal structures) as revealed by XANES analysis (Additional file 1: Table S1). TiO_2_ particles were mostly uniform in shape and size, ranging from approximately 200–300 nm in most samples (Table 1).

Matrix-assisted laser desorption/ionization mass spectrometry (MALDI-MS) analysis revealed the presence of organic copper (Cu) phthalocyanines pigments, amongst others (Additional file 1: Table S2). Presence of Cu is further verified by XRF analysis (Fig. 1a), which resolved Cu-phthalocyanine pigments with sizes of about 100 nm as well as big agglomerates in the micrometer range (Table 1, Additional file 1: Figure S1).

In all five skin and four lymph node samples of three donors analyzed via nano-XRF, Fe-Cr-Ni particles were observed in close relation to Ti particles (Fig. 1, Additional file 1: Figure S1). Their size varied from resolution limit of 1 pixel, which corresponds to 50 nm or smaller, up to the micrometer range. Hence, micrometer-sized particles also reach the lymph nodes. The chemical speciation of both Cr and Ni in these Fe particles was a mixture of metallic and oxidized elements as revealed by the XANES analysis (Fig. 1b, Additional file 1: Tables S3 and S4). Therefore, Fe particles more likely originate from steel particles than from Fe oxide pigments. It must be noted that the Ni concentration assessed by inductively coupled plasma (ICP)-MS was below the limit of detection in six out of 13 samples of tattooed donors and were only noticeably increased in three samples when compared to the controls (Additional file 1: Table S5). In contrast, synchrotron nano-XRF, with its very high sensitivity and high spatial resolution capability, can reveal high concentrations of small particles present in a restricted area of the skin. More specifically, it showed that all tissues exposed to tattoo pigments contained Fe-Cr-Ni steel particles.

### Steel particles are being abraded from the tattoo needle by TiO_2_

**Fig. 2:**
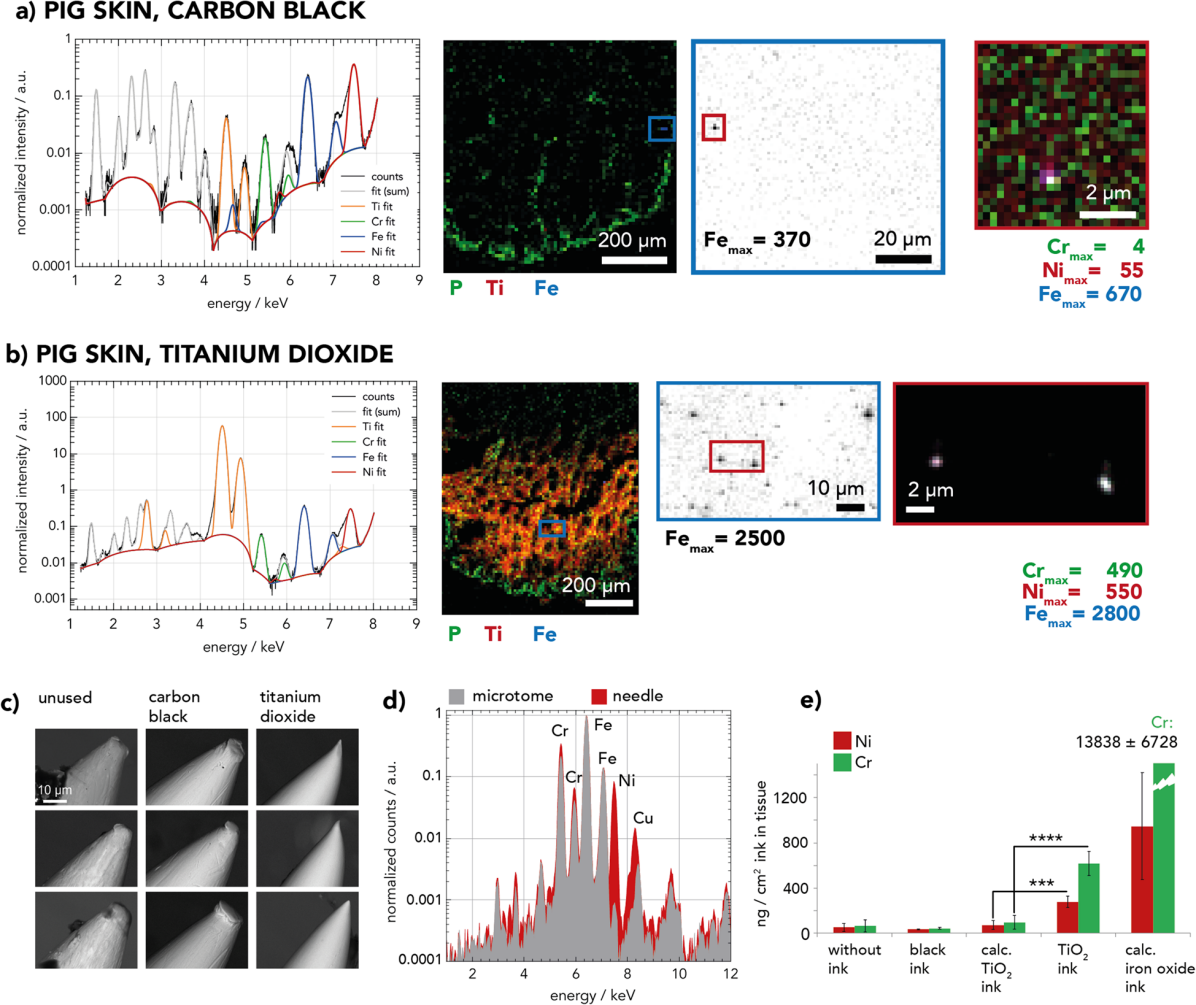
Nano-X-ray fluorescence (XRF) and scanning electron microscopy (SEM) analyses of pig skin tattooed with carbon black or titanium dioxide (TiO_2_) inks. **a**, **b** Zoom-in nano-XRF maps of tattooed pig skin sections recorded at beamline ID21 show a higher deposition of iron-chromium-nickel (Fe-Cr-Ni) particles with white TiO_2_ ink (**b**) than with carbon black (**a**). Maximal (max.) intensities of elements are given for each element. **c** SEM images of nine tattoo needles unused and after tattooing of pig skin with carbon black and TiO_2_ ink. Polished needle surfaces appear only after tattooing with TiO_2_ ink. **d** Table-top XRF spectra of tattoo needle and microtome knife. **e** Quantity of Ni and Cr per weight of tissue in pig skin after tattooing with carbon black, TiO_2_ or without (w/o) ink, analyzed by inductively coupled plasma mass spectrometry (ICP-MS) and estimated calc. TiO_2_ ink (see text for explanations). Worst case concentrations of highly contaminated brown iron oxide ink [9] were calculated in the same manner (calc. iron oxide ink). Asterisks indicate significances as identified by a two-way ANOVA with a Tukeys multiple comparison test using all tattooed samples and calc. TiO_2_ ink. Significant differences of pig skin tattooed with TiO_2_ ink compared to calc. TiO_2_ ink are displayed (*** = *p* < 0.001; **** = *p* < 0.0001). Data are displayed as mean of three distinct samples ± SD. Abbreviations: P = phosphorus

The steel debris found in human tissues alongside tattoos in the nano-XRF analysis may potentially derive from three sources: contaminated inks, contamination during sample preparation (wear from the microtome blades used for tissue sectioning), or wear from tattoo needles. We analyzed 50 tattoo inks from worldwide origins by means of nano-XRF that were either black, white or red and partially contained TiO_2_. None of the inks contained metallic Fe particles along with Ni and Cr contamination as the ones discovered in the skin and lymph node samples (data not presented). Furthermore, the microtome blades did not contain Ni, which excluded contamination from sample preparation (Fig. 2, Additional file 1: Table S6). However, all 12 tattoo needles analyzed contained 15–20% Cr and 6–9% Ni (Fig. 2d, Additional file 1: Table S6). To further determine the steel debris’ origin, we tattooed pig skin with either carbon black ink or TiO_2_ ink, with the latter known to have abrasive properties (Fig. 2). Both inks were assessed beforehand and were found not to contain steel particles (Additional file 1: Figure S2). The results show that the pig skin tattooed with TiO_2_ ink (Fig. 2b) contained by far more Fe-Cr-Ni particles than the skin tissue tattooed with carbon black (Fig. 2a).

Complementary scanning electron microscopy (SEM) images of the tattoo needle prior or after use furthermore revealed a completely polished needle after tattooing a skin surface as little as 2–3 cm^2^ with TiO_2_ ink (Fig. 2c). Nano-XRF and SEM analyses show similar metallic wear induced by TiO_2_ when tattooing pig skin with rotary and coil tattoo machines and corresponding needle equipment. The Ni and Cr deposition of steel particles was quantified by ICP-MS (Fig. 2e). The TiO_2_ ink showed minimal Ni and Cr background levels. The Ti counts per cm^2^ skin (tattooed with TiO_2_ ink) were compared to those of a known amount TiO_2_ ink to extrapolate the amount of ink in the skin (see Additional file 1: Figure S3). Ni and Cr concentrations in untreated skin and in TiO_2_ ink were then used to calculate the expected concentrations of these elements in TiO_2_ ink tattooed pig skin (see ‘calc. TiO_2_ ink’ in Fig. 2e). Both, concentrations of Ni and Cr were significantly higher upon tattooing (see TiO_2_ ink) compared to the calculated values. The additional elemental load deriving from abraded steel particles was calculated by subtracting the expected element levels (calc. TiO_2_ ink) from the mean quantified levels in tattooed pig skin (TiO_2_ ink) which were 206 ng/cm^2^ and 522 ng/cm^2^ for Ni and Cr, respectively (Additional file 1: Figure S3b). However, the additional Ni and Cr concentration inserted into skin by this steel wear particles is lower compared to the estimated deposition of these elements in skin upon usage of a highly contaminated brown ink (cf. calc. Iron oxide ink, Fig. 2e).

### Two types of Fe particles are present in allergic skin reaction

**Fig. 3:**
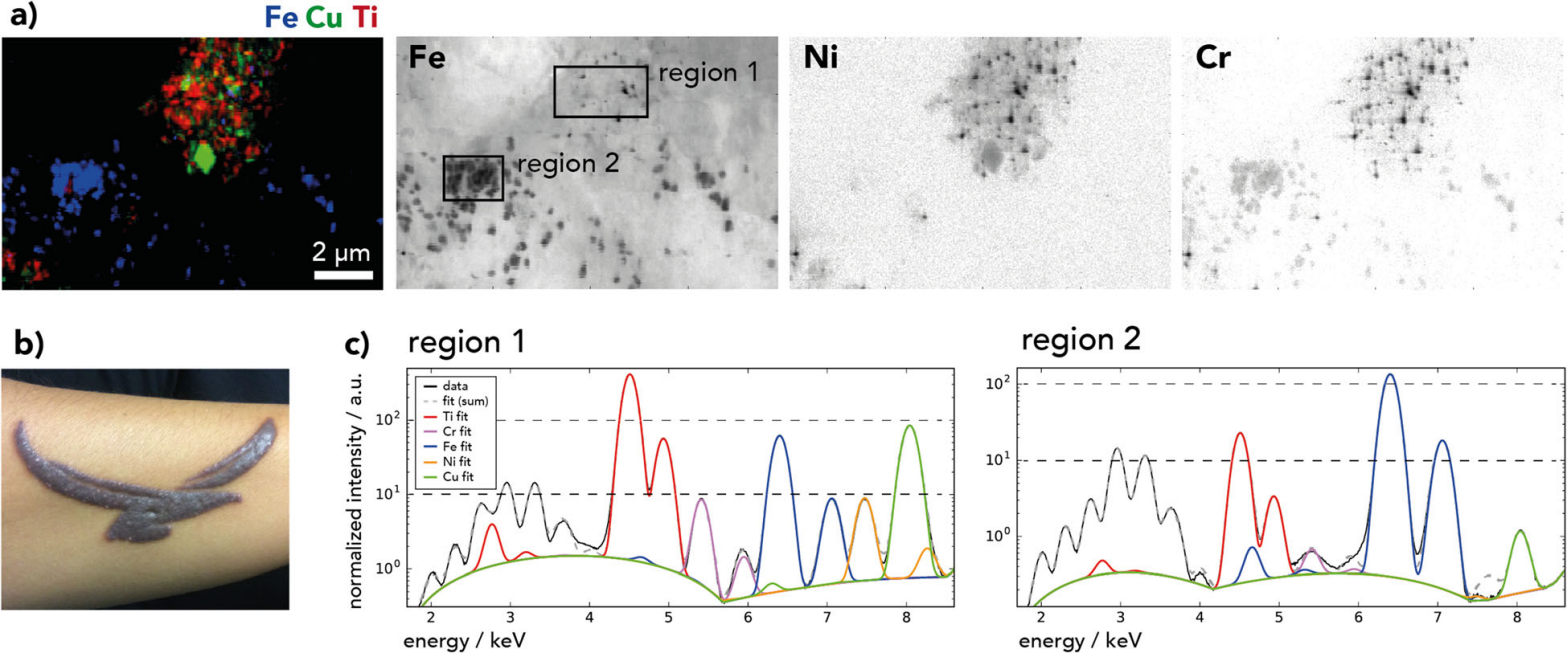
Allergic reaction to a red-brown tattoo of a patient sensitized to nickel. **a** Skin section nano-X-ray fluorescence (XRF) image recorded at beamline ID16B with distinct areas of iron (Fe) pigments (region 2) and smaller Fe-chromium-nickel (Fe-Cr-Ni) particles (region 1) in titanium (Ti)-rich regions. **b** Photography of the skin’s reaction to the tattoo before removal by dermabrasion. **c** Normalized XRF spectra in Fe pixels extracted from regions 1 and 2, as indicated in the Fe XRF image in (**a**). Abbreviations: Cu = copper

We also investigated the skin section of a patient who had experienced an allergic reaction to his tattoo (Fig. 3) and, as revealed by patch testing, was sensitized against Ni but not Cr. T-cell infiltration was verified by immunohistochemistry (Additional file 1: Figure S4). Organic pigment analysis by MALDI-MS revealed the presence of blue Cu-phthalocyanine which, as mentioned above, can be localized through high-resolution Cu nano-XRF maps (Fig. 3a, Additional file 1: Table S2).

In region 1, Ti and Cu are present as large particles and are likely part of a blue ink. Fe particles are much smaller and contain Ni and Cr (Fig. 3c, Table 1).

Region 2 presents a high concentration and more uniform distribution of Fe particles, but without Cu and Ti. The intensity of Cr is lower compared to region 1 and Ni is hardly detectable. Fe distribution is comparable to the one observed on nano-XRF maps of red iron oxide tattoo inks (Additional file 1: Figure S2). Therefore, Fe likely originates from red-brown Fe oxide pigments with a size of about 300–450 nm. The presence of a true Fe oxide pigment as a color-giving ingredient is supported by the high Fe concentration quantified by means of ICP-MS in the allergic skin sample (Additional file 1: Table S5).

**Fig. 4:**
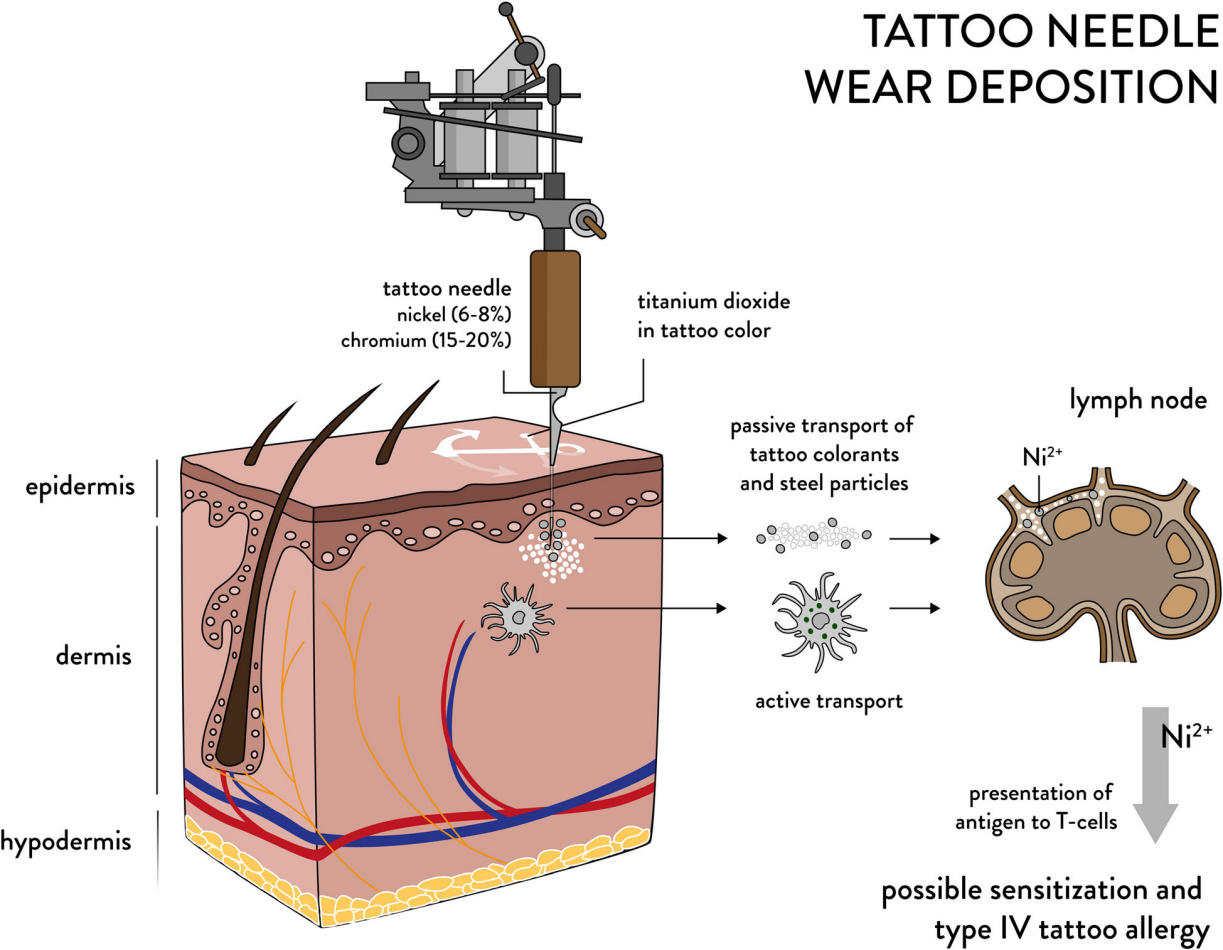
Tattoo needle wear biodistribution and supposed contribution to allergy formation. Nickel and chromium containing wear particles are abraded by titanium dioxide containing inks. The steel used to manufacture tattoo needles contains 6-8% nickel and 15-20% chromium. Pigments and wear particles are inserted into the dermal part of the skin. Both are passively and actively transported to the draining lymph nodes where Ni^2+^ ions as sensitizing species of nickel can be released. Antigen presentation to T-cells takes place in the lymph nodes as part of the adaptive immune response which is an obligatory process for sensitization and thus type IV allergy formation in the skin

In summary, the patient was tattooed with a self-mixed color deriving from two inks, which likely resulted in differing ink particle regions in the skin. The particular presence of Fe-Cr-Ni particles in the Ti rich regions can be assigned to putative steel wear particles from the tattoo needle (cf. Fig. 4). Hence, the skin of this patient contains two kinds of potential Ni sources – steel wear particles with high Ni concentrations and Fe oxide pigments with lower concentrations of Ni.

## Discussion

Our findings show that nano- and micrometer sized particles are abraded from tattoo needles when using TiO_2_-containing ink. These particles contain Ni and Cr and are permanently deposited in tattooed skin and are translocated to lymph nodes. Although the overall sample size was limited by the availability of specimens and the synchrotron beamtime, it is beyond doubt that the metal particles derive from the tattoo needle as result of pure mechanical stress. The particle deposition was additionally proven in pig skin and appeared only significant upon usage of TiO_2_ white ink but not with carbon black ink.

We also demonstrate that tattoo-derived particles larger than 2 μm are being translocated to the lymph nodes. We thus assume that a size threshold for particles transported toward the lymph nodes does not exist for the average tattoo particles of sizes below 100 nm to about 1 μm [14]. Since most of these wear particles are comparably small and reach down to the limit of resolution of 50 nm, biodistribution to other organs can be expected which is already known from human and animal studies [17, 18]. This study therefore provides the first proof that not only tattoo pigments but also abraded Fe-Cr-Ni steel particles are distributed toward the lymph nodes [13, 17, 19].

The tattoo reaction investigated in this article is classified as allergic type reaction. Tattoo allergies are characterized by non-infectious, chronic reactions with persistent reactions exceeding 3 months together with itching, swelling and dermal inflammation confined to one specific color inside the tattoo [20]. All these criteria were fulfilled with this patient.

Until now, the source of Ni and Cr in metal-related tattoo allergies was thought to be primarily contaminated Fe oxide pigment which commonly contains Ni, Cr, Cu or Co, amongst other elements [9]. However, we analyzed skin tissue from a patient who suffered from a tattoo-related allergic reaction and found both Fe oxide pigments as well as abraded steel particles in the inflamed skin. As the patient was sensitized to Ni, Ni allergy has likely caused the visible tattoo reaction.

Type IV Ni contact allergy is triggered by the Ni^2+^ ion in humans [21]. The Ni^2+^ ion may be released from the abraded steel particles through chemical alteration by reactive oxygen species inside the phagolysosomes of cells [22]. Hence, similar to Ni^2+^ release from steel implants or jewelry, the steel wear particles deriving from the tattoo needle can be sources of Ni^2+^ ion release [23–25]. Given the small size and hence larger surface-to-volume ratio of the wear particles, a comparably higher Ni^2+^ release is expected, which may then lead to sensitization and allergic reactions in the skin [23]. Similar to tattoo allergies, the number of metal allergy-related implant complications from abraded steel particles is low in comparison to the prevalence of cutaneous metal sensitization rates towards Ni in the general population [26, 27]. On the contrary, people with failed implants have a two to three times higher incidence of sensitization against metals. Thus, not every sensitized person will show a reaction to dermal depositions of these elements but nevertheless may be more likely to develop an adverse reaction.

With the evidence provided in this study arises the question of whether metallic wear from tattoo needles may indeed, just as Fe oxide pigments, play a role in allergic tattoo reactions. A connection between adverse tattoo effects, implant failure and also the use of TiO_2_ in tattoo inks has already been reported [28, 29]. Sensitization is also promoted by co-stimulating factors like inflammation, which may develop directly after tattooing due to the skin injury caused by the tattooing procedure or if infections occur simultaneously [30]. The impact of abraded particles from the tattoo needle and their potential role in metal-related tattoo skin allergies is yet unknown. Further investigations addressing a nickel sensitized cohort displaying tattoo allergy with and without tattoo needle wear due to TiO_2_ inks compared to allergies driven by iron oxide pigments alone will be, however, limited by the availability of such samples.

## Conclusion

In summary, the fact that all pigment and wear particles are deposited in lymph nodes calls for special attention to be placed on allergy development. Since the underlying substances causing tattoo allergies are barely known, tattoo needle wear containing chromium and nickel found in this investigation brings up a new source of sensitizers that need to be considered as additional source in allergy development. To date, neither additional nano-related effects nor a potential health impact of the utilized tattoo equipment are being considered in any tattoo legislation.

## Methods

### Human samples

Samples of tattooed skin areas and regional lymph nodes of five donors as well as skin and lymph node samples of two additional donors without any tattoos were taken *postmortem* at the Institute of Forensic Medicine at the Ludwig-Maximilians University of Munich (court-ordered autopsies with no additional cosmetic impairment to the skin). The experiments were performed according to the Helsinki Declaration of 1975. All samples were obtained anonymously without information on the donors’ disease status, causes of death or demographics. Hence, occupational or environmental exposure with high exposure with stainless steel or metallic particles is unknown. We selected specimens with tattoos other than black which are more likely to contain TiO_2_ and organic pigments.

The shave biopsy of the allergic patient was taken upon informed consent due to medical indication. The patient was tattooed with a self-made mixture of black and red ink in one session to achieve a brown color in 2014. The patient experienced immediate discomfort with additional swelling and itching with some delay of several weeks. After a year, the patient sought medical advice. The prescribed betamethasone cream applied twice a day only improved itching not swelling. The tattoo was removed in 2016. Tissue culture of removed skin was negative for mycobacteria. The patient showed a Ni (+++) patch test result with no reaction toward Cr in 2016. Removed tissue was received upon informed consent. Tissue samples were stored in plastic bags at − 20 °C directly after excision and further processed for analysis within a year. Subsamples were cut using a standard scalpel and frozen in TissueTek O.C.T. matrix (Sakura Finetek, Staufen, Germany) for cryo-microtome sectioning. Sections for XRF analyses at ID16B and ID21 were performed on 12–14 μm sections between two 4 μm Ultralene window films (Spex Sample Prep, Metuchen, NJ, USA). Sections were inactivated using a 4% formaldehyde buffer for 10 min and subsequently washed with deionized water (twice, 2–5 min) before being dried at room temperature.

### Tattooed pig skin samples

Abdominal pig skin was taken *postmortem* and stored at − 20 °C after hair was removed using an electric razor. Thawed skin was tattooed with a Cheyenne Hawk Thunder rotary tattoo machine (MT.Derm, Berlin, Germany) with various tattoo needles (cf. Additional file 1: Table S6) until an even color shade was achieved. Alternatively, a no-name coil tattoo machine was used. Tattooed specimens were prepared for microtome sectioning for XRF analysis as stated above. For ICP-MS analysis, skin pieces were cut out with a ceramic knife. Side lengths were measured and used to calculate the surface areas of the dissected skin squares.

### Synchrotron XRF

XRF analyses were carried out at the beamlines ID21 (micro-XRF) and ID16B (nano-XRF) at the European Synchrotron Radiation Facility (ESRF) in Grenoble, France, as previously described [31, 32]. For details see Additional file 1.

### XRF of microtome blades and tattoo needles

XRF analysis of 12 tattoo needles and 4 microtome knifes was carried out with a desktop Fischerscope X-ray XDV-SDD (Helmut Fischer GmbH Institut für Elektronik und Messtechnik, Sindelfingen, Germany). The instrument was equipped with an SDD detector with a 50 mm^2^ effective detector area and an aperture of 3 mm in diameter. The micro focus tube with tungsten target and beryllium window was set to 50 kV with a Ø 0.2 mm collimator. Each microtome knife as well as each tattoo needle was measured at four different positions for 30 s. For the microtome knife: two positions on the knife, two positions on the bulk; for the tattoo needle: two positions on the tips of the needles, two positions on the shaft of the needle.

### SEM analysis

SEM pictures of tattoo needle tips were recorded with a TM3030 tabletop microscope (Hitachi, Tokyo, Japan) at 15 kV accelerating voltage. Control needles were analyzed directly out of the package. Needles used for tattooing with either black or TiO_2_ ink were cleaned with isopropanol in an ultrasonic water bath (Sonorex digitec, Bandelin Electronic, Berlin, Germany) to remove residual tattoo ink.

### ICP-MS

For human tissue samples element analysis using ICP-MS was carried out as previously described [13]. For quantification of stainless steel wear, the method was altered to completely dissolve the steel particles. Therefore, 50–150 mg samples were digested with 6 ml HNO_3_ and 2 ml HCl in polytetrafluoroethylene vessels in a START 1500 microwave (MLS GmbH, Leutkirch, Germany). Samples were treated with 1000 W for 10 min at a temperature of 210 °C inside the reference vessel and 100 °C outside the protective shell. Afterwards, the temperature of the shell was raised to 150 °C for an additional 15 min. A cool-down time of 40 min was programmed. Full microwave digestion of steel wear was successfully tested by determination of the recovery from stainless steel nanoparticles (AISI 304 alloy, Fe/Cr18/Ni10, 45 μm from Goodfellow, Huntingdon, UK).

Samples were filled to 50 ml and subsequently analyzed with an iCAP Qc (Thermo Scientific, Waltham, MA, USA) with 10 sweeps per sample, a resolution of 0.1 u and a dwell time of 0.01 s in − 3 V kinetic energy discrimination mode. ^103^Rh was used as internal standard. ^52^Cr and ^60^Ni were monitored for quantitation. ^49^Ti counts were used to calculate relative ink concentrations in different samples. Limit of detection (LOD) and quantification (LOQ) were determined using the calibration curve method (DIN 32645). For Ni, 0.14 ppb was determined as LOD and 0.35 ppb as LOQ. LOD and LOQ for Cr were 0.08 ppb and 0.215 ppb, respectively.

### MALDI-MS

Organic pigments in the tissue samples have been identified using MALDI-MS as previously described [13]. Identification of pigments is based on the comparison of spectra to those of known pigment standards and must show corresponding molecular mass ions and other characteristic peaks.

### XANES analysis

Athena Demeter software [33] was used for XANES spectra fitting to standard substances. Where necessary, multiple spectra were averaged and normalized within the PyMCA software [34]. Spectra were normalized in 2nd order aligned to a standard spectrum, before using linear combination fitting. Fitting of all combinations was carried out with a maximum of four standards, all weighted between 0 and 1.

### Statistical analysis

Data from the quantification of Ni and Cr in pig skin were analyzed using the statistics software GraphPad Prism 6 (Graphpad Software Inc., La Jolla, CA, USA). A two-way ANOVA with Tukeys multiple comparison test was carried out to determine significant differences between the different groups. For both Ni and Cr ANOVA analyses degrees of freedom were two and three for columns and rows, respectively. The F ratios for the different treatments were 60.82 for Ni and 100.1 for Cr.

## Additional file

Additional file 1: Additional Methods. **Figure S1**. Nano-X-ray fluorescence (XRF) maps of four skin and three lymph node samples analyzed at ID16B. **Figure S2**. Nano-X-ray fluorescence (XRF) maps of selected inks analyzed at ID16B. **Figure S3**. Calculation of Ni and Cr contamination in pig skin and inks. **Figure S4**. T-cell infiltration in tattoo allergy sample. **Table S1**. Titanium XANES spectra of eight human skin and six lymph node samples as well as a skin allergy biopsy were fitted to pure anatase and rutile spectra of known standards. **Table S2**. MALDI-MS analysis of organic pigments in skin and lymph node samples. No pigments were found in the control samples. **Table S3**. Cr K-edge micro-XANES spectra of human skin and lymph node samples were fitted to spectra of known Cr standards. **Table S4**. Ni K-edge nano-XANES spectra of human skin and lymph node samples were fitted to known Ni standards. **Table S5**. ICP-MS analysis of elements in skin and lymph node samples. Increased values compared to skin or lymph node (LN) control samples are marked in bold. **Table S6**. Table-top X-ray fluorescence (XRF) analysis of microtome blades used for sample preparation and commercial tattoo needles. Tattoo needles analyzed derived from six different brands. Data are displayed as mean and standard deviation of *n* = 2 measurements. (DOCX 3000 kb)

## Data Availability

The XRF data sets and fitted maps generated and analyzed during the current study are available in the data dryad repository [35]. All other datasets used or analyzed during the current study are available from the corresponding author on reasonable request.

## Abbreviations

Co: Cobalt
Cr: Chromium
Cu: Copper
Fe: Iron
Hg: Mercury
ICP: Inductively coupled plasma
LOD: Limit of detection
LOQ: Limit of quantification
MALDI-MS: Matrix-assisted laser desorption/ionization mass spectrometry
Ni: Nickel
SEM: Scanning electron microscopy
TiO_2_: Titanium dioxide
XANES: X-ray absorption near-edge structure
XRF: X-ray fluorescence
