# Supplementary material for: Distribution of nickel and chromium containing particles from tattoo needle wear in humans and its possible impact on allergic reactions
Source: Part Fibre Toxicol. 2019 Aug 27;16:33. doi: 10.1186/s12989-019-0317-1 (PMC6710876; doi:10.1186/s12989-019-0317-1)
Supplement: Supplementary file 1 — Additional Methods. Figure S1. Nano-X-ray fluorescence (XRF) maps of four skin and three lymph node samples analyzed at ID16B. Figure S2. Nano-X-ray fluorescence (XRF) maps of selected inks analyzed at ID16B. Figure S3. Calculation of Ni and Cr contamination in pig skin and inks. Figure S4. T-cell infiltration in tattoo allergy sample. Table S1. Titanium XANES spectra of eight human skin and six lymph node samples as well as a skin allergy biopsy were fitted to pure anatase and rutile spectra of known standards. Table S2. MALDI-MS analysis of organic pigments in skin and lymph node samples. No pigments were found in the control samples. Table S3. Cr K-edge micro-XANES spectra of human skin and lymph node samples were fitted to spectra of known Cr standards. Table S4. Ni K-edge nano-XANES spectra of human skin and lymph node samples were fitted to known Ni standards. Table S5. ICP-MS analysis of elements in skin and lymph node samples. Increased values compared to skin or lymph node (LN) control samples are marked in bold. Table S6. Table-top X-ray fluorescence (XRF) analysis of microtome blades used for sample preparation and commercial tattoo needles. Tattoo needles analyzed derived from six different brands. Data are displayed as mean and standard deviation of n = 2 measurements. (DOCX 3000 kb) [file 12989_2019_317_MOESM1_ESM.docx]

ADDITIONAL FILE 1

Distribution of Nickel and Chromium Containing Particles from Tattoo Needle Wear in Humans and Its Possible Impact on Allergic Reactions

**Ines Schreiver^1,*,†^, Bernhard Hesse^2,3,*^, Christian Seim^3,4,5^, Hiram Castillo-Michel^2^, Lars Anklamm^6^, Julie Villanova^2^, Nadine Dreiack^1^, Adrien Lagrange^3,7^, Randolf Penning^8^, Christa De Cuyper^9^, Remi Tucoulou^2^, Wolfgang Bäumler^10^, Marine Cotte^2,11^ & Andreas Luch^1^**

^1^ German Federal Institute for Risk Assessment (BfR), Department of Chemical and Product Safety, Max-Dohrn-Strasse 8–10, 10589 Berlin, Germany.

^2^ The European Synchrotron, CS 40220, 38043 Grenoble Cedex 9, France.

^3^ Xploraytion GmbH, Bismarckstrasse 10–12, 10625 Berlin, Germany.

^4^ Physikalisch-Technische Bundesanstalt, Department of X-ray Spectrometry, Abbestrasse 2–12, 10587, Berlin, Germany.

^5^ Technical University Berlin, Institute for Optics and Atomic Physics, Hardenbergstrasse 36, 10623 Berlin, Germany.

^6^ Helmut Fischer GmbH Institut für Elektronik und Messtechnik, Industriestrasse 21, 71069 Sindelfingen Germany.

^7^ Technical University Berlin, Institute of Materials Science and Technologies, Strasse des 17. Juni 135, 10623 Berlin, Germany.

^8^ Ludwig-Maximilians University, Institute of Forensic Medicine, Nussbaumstrasse 26, 80336 Munich, Germany

^9^ Meiboomstraat 15, 8370 Blankenberge, Belgium.

^10^ University of Regensburg, Department of Dermatology, 93042 Regensburg, Germany.

^11^ Sorbonne University, Laboratory of Molecular and Structural Archaeology (LAMS), CNRS, UMR8220 Paris, France.

^*^These authors contributed equally to this work

**^†^Correspondence:** Dr. Ines Schreiver, Email: [ines.schreiver@bfr.bund.de](mailto:ines.schreiver@bfr.bund.de), Tel: +49 30 18412 27800.

**Abbreviations used**: **Cr :** chromium; **Hg :** mercury; **Co :** cobalt; **Ni :** nickel; **XRF :** X-ray fluorescence; **Fe :** iron; **XANES :** X-ray absorption near-edge structure; **TiO_2_ :** titanium dioxide; **Cu :** copper; **MALDI-MS :** matrix-assisted laser desorption/ionization mass spectrometry; **SEM :** scanning electron microscopy; **ICP :** inductively coupled plasma; **LOD :** limit of detection; **LOQ :** limit of quantification

**Additional Methods**

**Synchrotron XRF**

Human skin and lymph node samples were investigated at ID16B using an energy of 9.5 keV. Acquisition time per point was 100 ms. The pixel size for collecting the XRF maps was adjusted for the regions of interest and varied from 0.05 μm to 1 μm.

For collecting Ni K-edge XANES spectra at ID16B, the energy of the incoming beam was scanned from 8.3 to 8.5 keV in increments of 0.5 – 5 eV, with acquisition times of 100 – 200 ms per energy. Depending on the concentration of the probed region, up to seven XANES spectra were collected per sample and were subsequently averaged. 2D XANES maps were also collected and average XANES spectra were calculated over multiple pixels.

Cr- and Ti-edge XANES were obtained at ID21. For collecting Cr K-edge XANES spectra, the energy of the incoming beam was scanned from 5.96 to 6.1 keV in increments of 0.5 – 1.5 eV with acquisition times of 100 ms per energy. Depending on the concentration of the probed region, between one and five XANES spectra were collected per point and up to five different points were analyzed per sample and were subsequently averaged. For collecting Ti K-edge XANES spectra, the energy of the incoming beam was scanned from 4.95 to 5.1 keV in increments of 0.5 – 5 eV, with acquisition times of 100 ms per energy. Depending on the concentration of the probed region, up to 12 XANES spectra were collected per sample and were subsequently averaged.

Ink samples were analyzed at ID16B with a 17.5 keV pink multibunch beam and acquiring XRF maps with high resolution 0.05 x 0.05 µm^2^ and 100 ms per point.

Pig skin sections were analyzed at ID21. Energy was set to 8.4 keV with 2.0 x 10^9^ flux (photons/s), reduced with an 30 μm aluminum attenuator to minimize dead time. The beam was focused down to 0.45 x 0.8 µm² (vertical × horizontal). Acquisition time per point was 100 ms. Despite the use of an attenuator, the high element concentrations sometimes lead to SDD saturation visible in the XRF spectra. In order to be sensitive enough for low abundant elements, we refrained from using thicker attenuators. The pixel size for collecting the XRF maps was adjusted to the regions of interest and varied from 0.25 μm to 10 μm.

**Additional Figures**

**Figure S1**. **Nano-X-ray fluorescence (XRF) maps of four skin and three lymph node samples analyzed at ID16B.** Spectra are averaged over the full map area. Skin and lymph node nano-XRF maps of donor 1 are depicted in Fig. 1 in the main manuscript. All maps are displayed in log scale. Abbreviations: Ti = titanium; Cr = chromium; Fe = iron; Ni = nickel; Cu = copper; LN = lymph node.


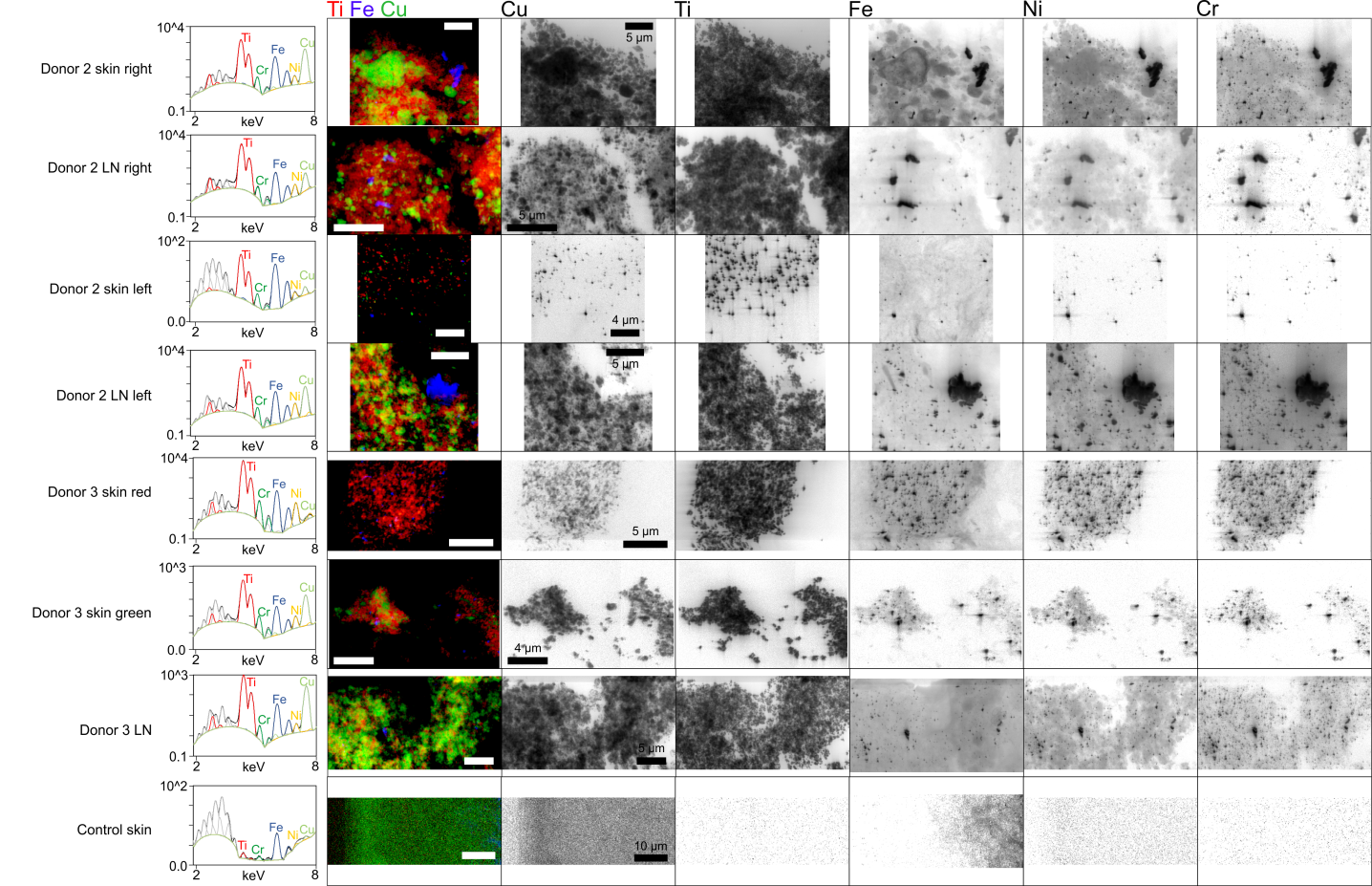


**Figure S2. Nano-X-ray fluorescence (XRF) maps of selected inks analyzed at ID16B.** Spectra are averaged over the full map area. All maps are displayed in log scale. Detector saturation leads to false nickel (Ni) signals in log maps of titanium dioxide (TiO_2_) ink and red ink 2 (cf. spectra with no Ni). We refrained from using more than 50 µm aluminum attenuators to keep sensitivity for low abundant elements. Abbreviations: S = sulfur; Cl = chlorine; Ar = argon; K = potassium; Ca = calcium; Cr = chromium; Fe = iron; Cu = copper; Zn = zinc; As = arsenic; Se = selenium; Br = bromine; Kr = krypton.


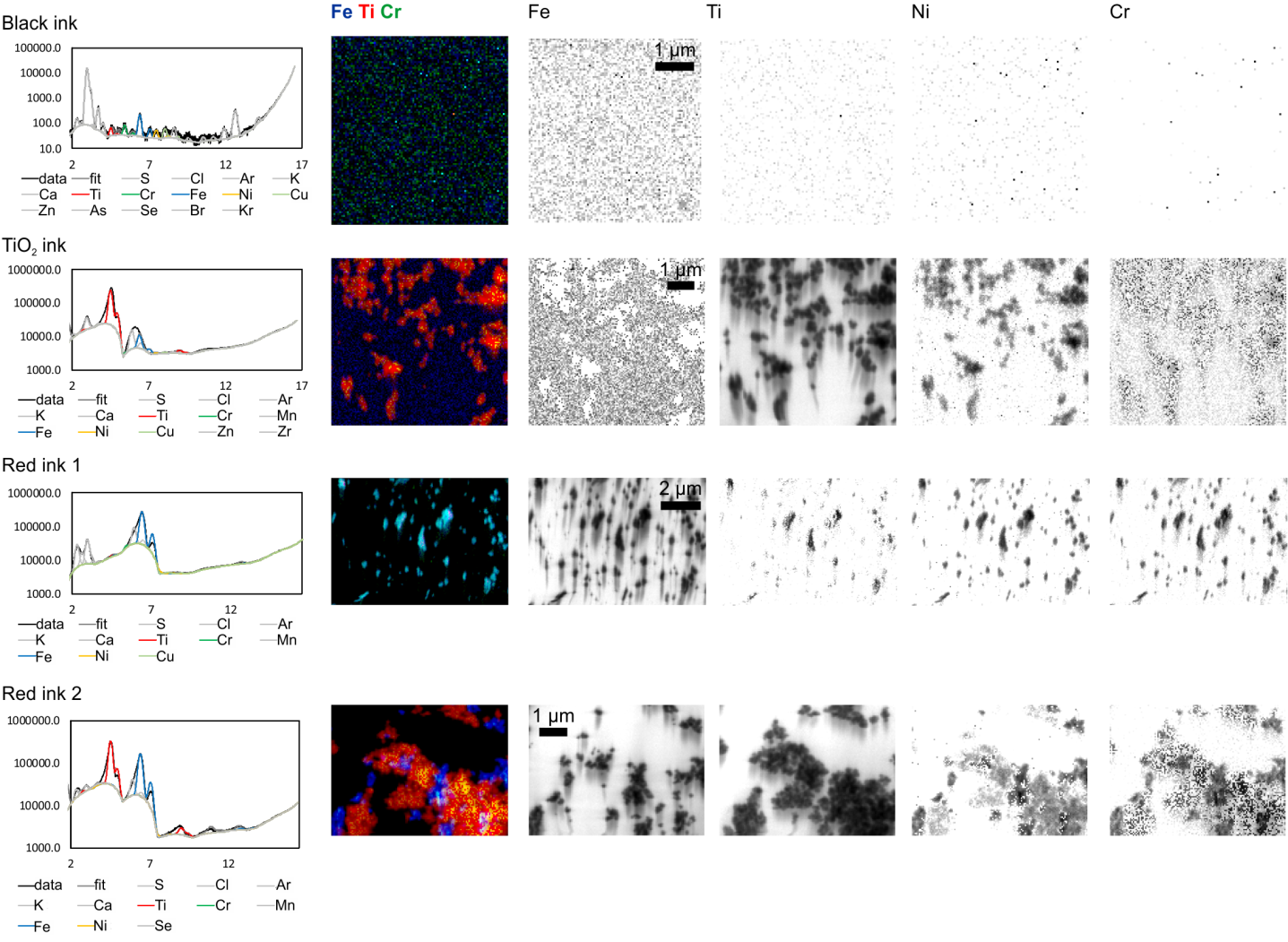
**Figure S3. Calculation of Ni and Cr contamination in pig skin and inks.** A) Scheme for calculation of Ni and Cr introduced by tattoo needles and iron oxide inks. B) Quantification (ICP-MS) and calculated values of Ni and Cr in pig skin. Abbreviations: Ti = titanium; w/o = without; TiO_2_ = titanium dioxide; Ni = nickel; Cr = chromium; calc. = calculated.

**
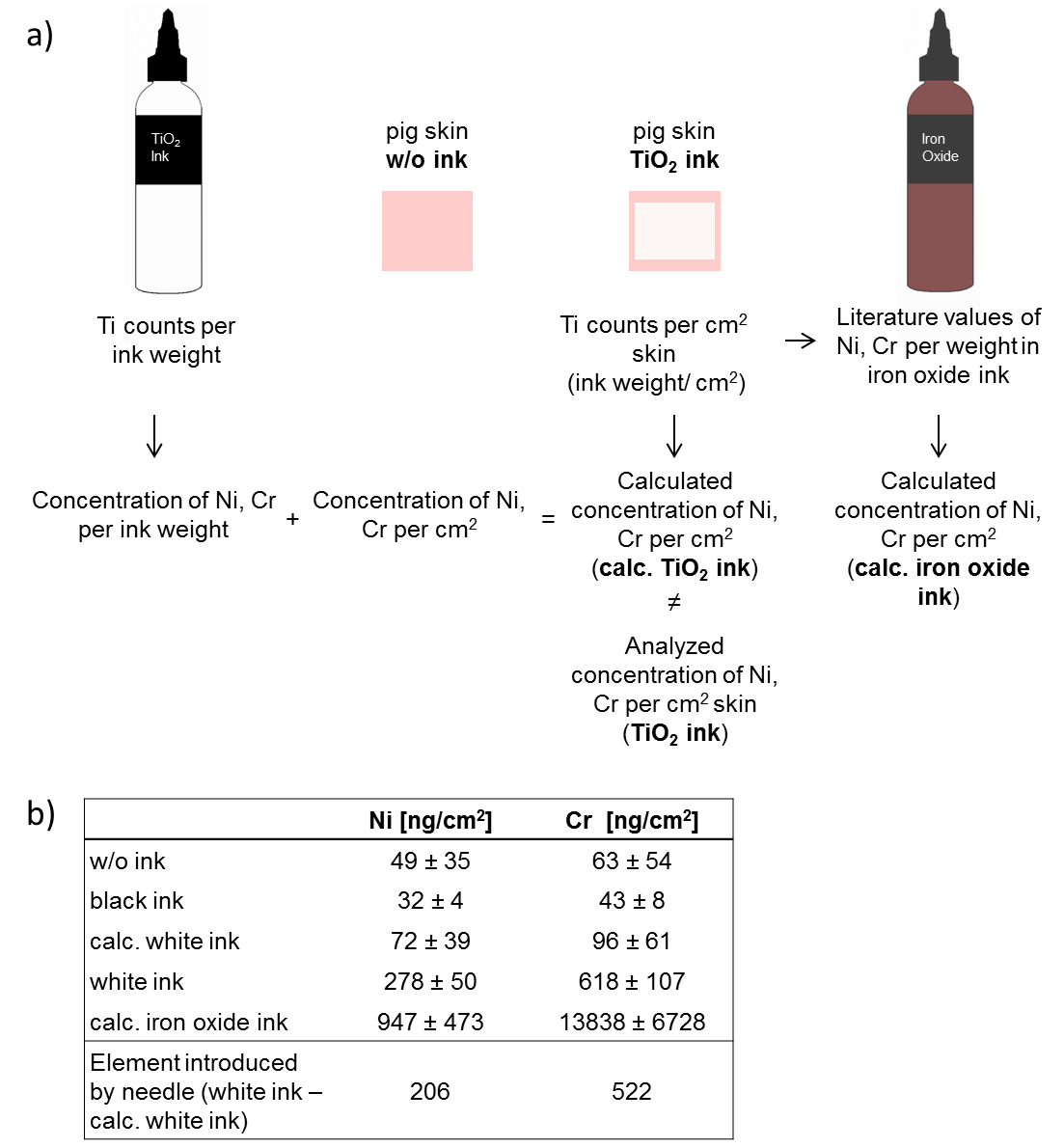
**

**Figure S4. T-cell infiltration in tattoo allergy sample.** T-cell infiltration in the inflamed skin of the allergic tattoo reaction was verified by CD3 staining. Cell nuclei were stained with DAPI. The tattoo pigment in the skin showed red autofluorescence (black spots in bright field).

**
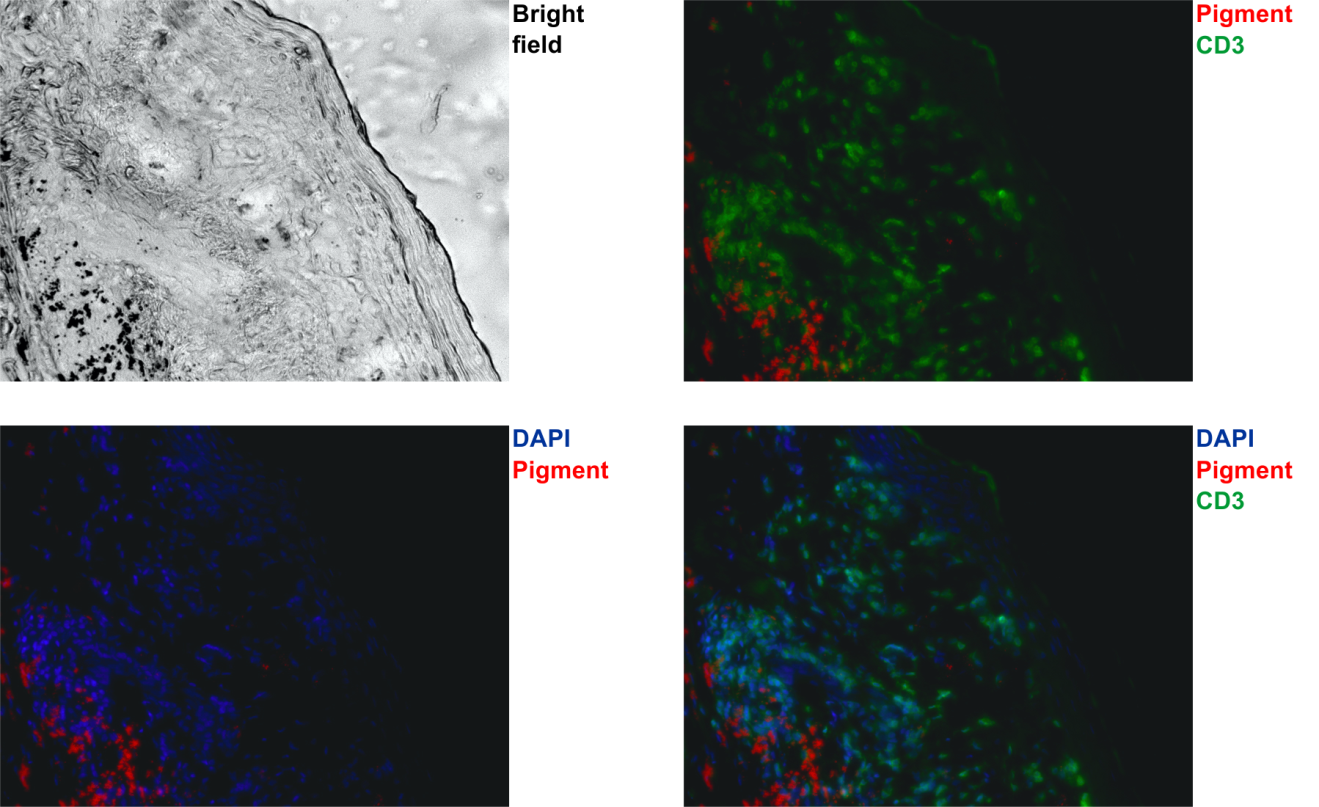
**

**Additional Tables**

**Table S1.** **Titanium XANES spectra of eight human skin and six lymph node samples as well as a skin allergy biopsy were fitted to pure anatase and rutile spectra of known standards**

|  | **anatase** | **rutile** | **R-factor** | **Chi-square** |
| --- | --- | --- | --- | --- |
| Donor 1 skin, map 1 | 0.0% | 100.0% | 0.003 | 0.045 |
| Donor 1 skin, map 2 | 93.5% | 6.5% | 0.001 | 0.022 |
| Donor 1 LN | 71.0% | 29.0% | 0.002 | 0.032 |
| Donor 2 skin left | 66.6% | 33.5% | 0.003 | 0.038 |
| Donor 2 LN left | 11.6% | 88.4% | 0.002 | 0.028 |
| Donor 2 skin right | No XANES | | | |
| Donor 2 LN right | No XANES | | | |
| Donor 3 skin green | 0.0% | 100.0% | 0.007 | 0.103 |
| Donor 3 skin red | No XANES | | | |
| Donor 3 LN | 0.0% | 100.0% | 0.005 | 0.073 |
| Donor 4 skin^1^ | 4.9% | 95.1% | 0.002 | 0.036 |
| Donor 4 LN^1^ | 2.4% | 97.6% | 0.001 | 0.017 |
| Donor 5 skin^1^ | No XANES | | | |
| Donor 5 LN^1^ | 93.6% | 3.5% | 0.002 | 0.050 |
| Allergy biopsy | 8.1% | 91.9% | 0.020 | 0.378 |

Abbreviations: Ti = titanium; XANES = X-ray absorption near edge structure; LN = lymph node.

^1^additional samples not investigated by nano-X-ray fluorescence

**Table S2. MALDI-MS analysis of organic pigments in skin and lymph node samples. No pigments were found in the control samples**

|  | **color** | **body region** | **identified pigment(s)** | **m/z of molecular ion** |
| --- | --- | --- | --- | --- |
| Donor 1 skin | black, red, green | arm, right | P.R.170 | 477 [M+H]+ |
| Donor 1 LN | dark | armpit, right | - |  |
| Donor 2 skin right | green | right | not determined |  |
| Donor 2 LN right | black, green | armpit, right | P.B.15 | 575 [M]+ |
| Donor 2 skin left | red | left | P.R.170 | 477 [M+H]+ |
| Donor 2 LN left | black, green | armpit, left | P.G.7, P.B.15 | 1126.5 [M]+, 575 [M]+ |
| Donor 3 skin | red | right | not determined |  |
| Donor 3 skin | green | right | P.G.7, P.B.15 | 1126.5 [M]+, 575 [M]+ |
| Donor 3 LN | black, green | right | P.G.7, P.B.15 | 1126.5 [M]+, 575 [M]+ |
| Donor 4 skin^1^ | yellow | left | not determined |  |
| Donor 4 LN^1^ | dark | left | P.G.7, P.B.15 | 1126.5 [M]+, 575 [M]+ |
| Donor 5 skin^1^ | red | arm, right | not determined |  |
| Donor 5 LN^1^ | red | armpit, right | - |  |
| Allergy biopsy | red, brown | arm | P.B.15 | 575 [M]+ |

Pigment identity: P.R.170 = C.I.12475; P.B.15 = C.I.74160; P.G.7 = C.I.74260.

Abbreviation: MALDI-MS = matrix-assisted laser desorption/ionization mass spectrometry; P.B. = pigment blue; P.R. = pigment red; P.G. = pigment green; M = molecular mass ion; M+H = protonated molecular mass ion.

^1^additional samples not investigated by nano-X-ray fluorescence

**Table S3. Cr K-edge micro-XANES spectra of human skin and lymph node samples were fitted to spectra of known Cr standards**

|  | **Cr(III)OH** | **metal Cr** | **Cr(VI)** | **R-factor** | **Chi-square** |
| --- | --- | --- | --- | --- | --- |
| Donor 1 skin, map 1 | 47.9% | 47.4% | 0.0% | 0.005 | 0.084 |
| Donor 1 skin, map 2 | 45.4% | 47.4% | 0.0% | 0.004 | 0.057 |
| Donor 1 LN | 66.4% | 36.1% | 0.0% | 0.009 | 0.169 |
| Donor 2 skin left | 53.5% | 46.6% | 0.0% | 0.016 | 0.293 |
| Donor 2 LN left | 57.3% | 42.9% | 0.0% | 0.014 | 0.270 |
| Donor 2 skin right | 77.0% | 30.3% | 0.0% | 0.017 | 0.341 |
| Donor 2 LN right | No XANES | | | | |
| Donor 3 skin red | 68.5% | 32.4% | 0.0% | 0.008 | 0.145 |
| Donor 3 skin green | 56.3% | 41.5% | 0.0% | 0.006 | 0.108 |
| Donor 3 LN | 62.0% | 39.8% | 0.0% | 0.013 | 0.245 |
| Donor 4 skin^1^ | low Cr | | | | |
| Donor 4 LN^1^ | 59.3% | 38.5% | 0.0% | 0.005 | 0.095 |
| Donor 5 skin^1^ | No XANES | | | | |
| Donor 5 LN^1^ | 47.2% | 50.6% | 0.2% | 0.010 | 0.194 |

Abbreviations: Cr = chromium; XANES = X-ray absorption near edge structure; LN = lymph node; Cr(VI) = hexavalent sodium dichromate; Cr(III)OH = trivalent chromium hydroxide.

^1^additional samples not investigated by nano-X-ray fluorescence

**Table S4.** **Ni K-edge nano-XANES spectra of human skin and lymph node samples were fitted to known Ni standards**

|  | **Ni sulphate** | **Ni acetate** | **Ni metal** | **Ni(OH)_2_** | **R-factor** | **Chi-square** |
| --- | --- | --- | --- | --- | --- | --- |
| Donor 1 skin, map 1 | 12.1% | - | 82.2% | 5.1% | 0.009 | 0.162 |
| Donor 1 skin, map 2 | 13.2% | - | 78.1% | 9.8% | 0.008 | 0.108 |
| Donor 1 LN | 8.4% | - | 83.8% | 6.8% | 0.021 | 0.344 |
| Donor 2 skin left | 27.6% | - | 69.3% | 4.9% | 0.014 | 0.275 |
| Donor 2 LN left | 16.6% | - | 79.1% | 5.0% | 0.008 | 0.155 |
| Donor 2 skin right | No XANES | | | | | |
| Donor 2 LN right | No XANES | | | | | |
| Donor 3 skin green | 15.9% | 15.9% | 68.5% | - | 0.036 | 0.543 |
| Donor 3 skin red | 17.6% | - | 68.2% | 13.6% | 0.005 | 0.069 |
| Donor 3 LN | 5.5% | - | 87.5% | 7.7% | 0.008 | 0.119 |
| Donor 4 skin^1^ | No XANES | | | | | |
| Donor 4 LN^1^ | No XANES | | | | | |
| Donor 5 skin^1^ | No XANES | | | | | |
| Donor 5 LN^1^ | No XANES | | | | | |

Abbreviations: Ni = nickel; XANES = X-ray absorption near edge structure; LN = lymph node.

^1^Additional samples not investigated by nano-X-ray fluorescence

^2^Neither Ni disulphide nor Ni carbonate were detected in the inks, although they were included as the standard spectra during the fitting procedure

**Table S5.** **ICP-MS analysis of elements in skin and lymph node samples. Increased values compared to skin or lymph node (LN) control samples are marked in bold**

| **[ppm]** | **color** | **Cu** | **Cr** | **Fe** | **Ni** | **other elements**  **>20,000 count** | **other elements**  **<20,000 count** |
| --- | --- | --- | --- | --- | --- | --- | --- |
| Donor 1 skin | black, red, green | 3.6 | 0.6 | 17.2 | 0.2 | Ti | Zn, Ba, Pb |
| Donor 1 LN | dark | <LOQ | <LOQ | 90.4 | <LOQ |  | Ti, Zn, Ba |
| Donor 2 skin left | green | **8.1** | <LOQ | 8.8 | <LOQ | Ti | Cr, Zn |
| Donor 2 LN left | black, green | **4.2** | 0.6 | 24.2 | 0.3 | Ti | Cr, Zn |
| Donor 2 skin right | red | <LOQ | <LOQ | 1.3 | <LOQ |  | Ti, Cr |
| Donor 2 LN right | black, green | **31.3** | **4.8** | 52.8 | **2.3** | Ti | Cr, Zn |
| Donor 3 skin | red | 0.7 | **6.8** | 41.8 | 0.7 |  | Ti |
| Donor 3 skin | green | 1.3 | 0.3 | 5.5 | 0.1 |  | Ti, Zn |
| Donor 3 LN | black, green | **48.2** | **4.0** | 40.0 | **1.7** | Ti | Zn |
| Donor 4 skin^1^ | yellow | <LOQ | <LOQ | 1.1 | <LOQ |  | Ti, Zn |
| Donor 4 LN^1^ | dark | 12.8 | <LOQ | 9.2 | <LOQ | Ti | Zn, Hg |
| Donor 5 skin^1^ | red | <LOQ | <LOQ | 2.9 | <LOQ |  |  |
| Donor 5 LN^1^ | red | 0.8 | 1.2 | **107.8** | 0.5 | Ti | Zn, Ba |
| Allergy biopsy | red, brown | **7.5** | **7.4** | **537.1** | **2.9** | Ti, Mn, Zn | Ba |
| Control skin 1 | - | 1.4 | 0.2 | 35.6 | 0.1 |  | Pb |
| Control LN 1 | - | 13.0 | 0.3 | 64.4 | 1.1 |  |  |
| Control skin 2 | - | 1.4 | 0.6 | 37.6 | 0.2 |  |  |
| Control LN 2 | - | 2.5 | 0.1 | 74.7 | 0.1 |  | Zn |
| Literature values |  |  |  |  |  |  |  |
| skin | - | 0.35^2^ |  |  |  |  |  |
| LN | - |  | 8.2^4^ | 1800^4^ | 0.28^3^ 3.7^4^ |  |  |

Abbreviations: ppm = parts per million, Cu = copper; Cr = chromium; Fe = iron; Ni = nickel; Ti = titanium; Zn = zinc; Hg = mercury; Ba = barium; Pb = lead.

^1^additional samples not investigated by nano-X-ray fluorescence

^2^ = Saltzman (1), wet basis, average from 21 male cadavers; ^3^ = Rezuke (2), tissue dry weight, average from 3 cadavers; ^4^ = Teraoka (3), tissue dry weight, average from 12 male cadavers.

**Table S6.** **Table-top X-ray fluorescence (XRF) analysis of microtome blades used for sample preparation and commercial tattoo needles. Tattoo needles analyzed derived from six different brands. Data are displayed as mean and standard deviation of n=2 measurements.**

| **Elements in %** |  | | **Cr** | | | **Mn** | | | **Fe** | | | **Ni** | | | **Cu** | | |
| --- | --- | --- | --- | --- | --- | --- | --- | --- | --- | --- | --- | --- | --- | --- | --- | --- | --- |
| Blade I | |  | 0.4 | ± | 0.0 | 0.3 | ± | 0.0 | 99.3 | ± | 0.1 | 0.0 | ± | 0.0 | 0.0 | ± | 0.0 |
| Blade II | |  | 12.2 | ± | 0.3 | 0.8 | ± | 0.1 | 87.0 | ± | 0.2 | 0.0 | ± | 0.0 | 0.0 | ± | 0.0 |
| Blade III | |  | 13.1 | ± | 0.5 | 0.8 | ± | 0.0 | 86.0 | ± | 0.5 | 0.1 | ± | 0.0 | 0.0 | ± | 0.0 |
| Blade IV | |  | 12.7 | ± | 0.2 | 0.8 | ± | 0.0 | 86.4 | ± | 0.2 | 0.1 | ± | 0.0 | 0.0 | ± | 0.0 |
| Coil needle (35/9RLLT) | | tip | 16.0 | ± | 0.3 | 2.4 | ± | 0.1 | 72.7 | ± | 0.3 | 6.6 | ± | 0.0 | 2.2 | ± | 0.1 |
|  | | side | 15.5 | ± | 0.1 | 2.4 | ± | 0.0 | 73.3 | ± | 0.1 | 6.4 | ± | 0.0 | 2.2 | ± | 0.0 |
| Coil needle (1215 MGL) | | tip | 19.0 | ± | 1.1 | 1.1 | ± | 0.1 | 70.9 | ± | 1.2 | 8.4 | ± | 0.1 | 0.4 | ± | 0.2 |
|  | | side | 17.9 | ± | 0.2 | 1.3 | ± | 0.0 | 71.9 | ± | 0.1 | 8.4 | ± | 0.1 | 0.4 | ± | 0.0 |
| Rotary needle (13-Magnum) | | tip | 19.2 | ± | 0.3 | 1.1 | ± | 0.2 | 70.5 | ± | 0.1 | 8.4 | ± | 0.1 | 0.6 | ± | 0.0 |
|  | | side | 18.4 | ± | 0.1 | 1.3 | ± | 0.0 | 71.4 | ± | 0.1 | 8.2 | ± | 0.1 | 0.6 | ± | 0.0 |
| Rotary needle (17-Magnum) ^1^ | | tip | 19.4 | ± | 0.0 | 0.5 | ± | 0.0 | 70.7 | ± | 0.0 | 8.5 | ± | 0.0 | 0.7 | ± | 0.1 |
|  | | side | 17.5 | ± | 0.1 | 0.8 | ± | 0.1 | 72.9 | ± | 0.2 | 8.2 | ± | 0.1 | 0.5 | ± | 0.0 |
| Rotary needle (23-Magnum) | | tip | 17.8 | ± | 0.1 | 2.2 | ± | 0.1 | 70.6 | ± | 0.5 | 6.6 | ± | 0.3 | 2.5 | ± | 0.2 |
|  | | side | 15.7 | ± | 0.2 | 2.6 | ± | 0.0 | 72.9 | ± | 0.1 | 6.4 | ± | 0.2 | 2.3 | ± | 0.1 |
| Rotary needle (5RL) | | tip | 18.2 | ± | 0.4 | 1.2 | ± | 0.1 | 71.5 | ± | 0.4 | 8.7 | ± | 0.1 | 0.3 | ± | 0.0 |
|  | | side | 17.5 | ± | 0.1 | 1.2 | ± | 0.0 | 72.4 | ± | 0.1 | 8.5 | ± | 0.1 | 0.3 | ± | 0.0 |
| Rotary needle (17MM) | | tip | 19.0 | ± | 0.3 | 1.4 | ± | 0.1 | 70.7 | ± | 0.3 | 8.4 | ± | 0.1 | 0.4 | ± | 0.1 |
|  | | side | 17.3 | ± | 0.0 | 1.6 | ± | 0.0 | 72.5 | ± | 0.1 | 8.2 | ± | 0.0 | 0.4 | ± | 0.1 |
| Coil needle (1223 MGL) | | tip | 20.0 | ± | 0.1 | 0.9 | ± | 0.1 | 69.7 | ± | 0.0 | 8.6 | ± | 0.1 | 0.5 | ± | 0.0 |
|  | | side | 18.0 | ± | 0.2 | 1.2 | ± | 0.0 | 71.9 | ± | 0.2 | 8.4 | ± | 0.1 | 0.4 | ± | 0.0 |
| Coil needle (1209FLL) | | tip | 19.3 | ± | 0.1 | 0.9 | ± | 0.1 | 70.8 | ± | 0.2 | 8.5 | ± | 0.1 | 0.4 | ± | 0.1 |
|  | | side | 17.9 | ± | 0.0 | 1.3 | ± | 0.1 | 71.9 | ± | 0.3 | 8.5 | ± | 0.0 | 0.4 | ± | 0.1 |
| Coil needle (1209RSL) | | tip | 16.3 | ± | 0.8 | 2.4 | ± | 0.2 | 72.7 | ± | 0.6 | 6.6 | ± | 0.3 | 2.0 | ± | 0.3 |
|  | | side | 15.7 | ± | 0.1 | 2.6 | ± | 0.1 | 73.3 | ± | 0.1 | 6.1 | ± | 0.1 | 2.2 | ± | 0.0 |
| Coil needle (1209RL) | | tip | 18.5 | ± | 0.4 | 1.1 | ± | 0.0 | 71.6 | ± | 0.3 | 8.6 | ± | 0.2 | 0.2 | ± | 0.0 |
|  | | side | 17.9 | ± | 0.0 | 1.3 | ± | 0.1 | 72.3 | ± | 0.3 | 8.3 | ± | 0.1 | 0.2 | ± | 0.1 |
| Coil needle (15M1 35) | | tip | 17.4 | ± | 0.1 | 1.4 | ± | 0.1 | 71.9 | ± | 0.2 | 8.8 | ± | 0.2 | 0.5 | ± | 0.0 |
|  | | side | 18.0 | ± | 0.1 | 1.3 | ± | 0.0 | 71.6 | ± | 0.1 | 8.5 | ± | 0.1 | 0.5 | ± | 0.1 |
| Coil needle (1205 M1) | | tip | 17.8 | ± | 0.4 | 1.0 | ± | 0.4 | 72.1 | ± | 0.5 | 9.0 | ± | 0.6 | 0.2 | ± | 0.1 |
|  | | side | 18.0 | ± | 0.3 | 0.6 | ± | 0.1 | 72.7 | ± | 0.3 | 8.3 | ± | 0.1 | 0.2 | ± | 0.0 |

Abbreviations: XRF = X-ray fluorescence; Cr = chromium; Mn = manganese; Fe = iron; Ni = nickel; Cu = copper.

^1^used for metallic wear analysis in pig skin (**Fig. 2**)

**References**

1. Saltzman BE, Gross SB, Yeager DW, Meiners BG, Gartside PS. Total body burdens and tissue concentrations of lead, cadmium, copper, zinc, and ash in 55 human cadavers. Environ Res. 1990;52:126-45.

2. Rezuke WN, Knight JA, Sunderman FW. Reference values for nickel concentrations in human tissues and bile. Am J Ind Med. 1987;11:419-26.

3. Teraoka H. Distribution of 24 elements in the internal organs of normal males and the metallic workers in Japan. Arch Environ Health. 1981;36(4):155-65.
